# Biochemical Characterization of a New Oligoalginate Lyase and Its Biotechnological Application in *Laminaria japonica* Degradation

**DOI:** 10.3389/fmicb.2020.00316

**Published:** 2020-03-10

**Authors:** Shangyong Li, Linna Wang, Samil Jung, Beom Suk Lee, Ningning He, Myeong-Sok Lee

**Affiliations:** ^1^School of Basic Medicine, Qingdao University, Qingdao, China; ^2^Molecular Cancer Biology Laboratory, Cellular Heterogeneity Research Center, Department of Biosystem, Sookmyung Women’s University, Seoul, South Korea; ^3^Yellow Sea Fisheries Research Institute, Chinese Academy of Fishery Sciences, Qingdao, China

**Keywords:** oligoalginate lyase, substrate specificity, alginate monomers, *Laminaria japonica*, *Vibrio* sp. SY01

## Abstract

Oligoalginate lyases catalyze the degradation of alginate polymers and oligomers into monomers, a prerequisite for biotechnological utilizing alginate. In this study, we report the cloning, expression and biochemical characterization of a new polysaccharide lyase (PL) family 17 oligoalginate lyase, OalV17, from the marine bacterium *Vibrio* sp. SY01. The recombinant OalV17 showed metal ion independent and detergent resistant properties. Furthermore, OalV17 is an exo-type enzyme that yields alginate monomers as the main product and recognizes alginate disaccharides as the minimal substrate. Site-directed mutagenesis followed by kinetic analysis indicates that the residue Arg^231^ plays a key role in substrate specificity. Furthermore, a rapid and efficient alginate monomer-producing method was developed directly from *Laminaria japonica*. These results suggest that OalV17 is a potential candidate for saccharification of alginate.

## Introduction

As the world energy demand continues to rise, macro-algae have been regarded as a renewable source for producing biofuels (Singh et al., 2011). Due to strong demand in the food industry and an increasing interest in the bioethanol and commodity chemical production sectors, the marine biomass market is predicted to reach $22 billion USD by 2024 (Razeghifard, 2013; Vuoristo et al., 2019). Brown seaweed, particularly *Laminaria japonica*, has attracted increasing attention due to its fast growth, high carbohydrate content and lack of lignin (Lipinska et al., 2019). Alginate, the most abundant and important carbohydrate in the cell wall of brown seaweed, accounts for 22–44% of its dry weight (Senturk Parreidt et al., 2018). Although bioethanol production from marine biomass-derived carbohydrates such as mannitol or laminarin has been reported, ethanol production from algae presents several technical difficulties, including the lack of efficient biotechnology and ethanologenic microbe to produce ethanol from alginate (alginic acid) (Horn et al., 2000; Yarden and Sliwkowski, 2001; Liu et al., 2019). Meanwhile, alginate are already oxidized carbohydrate and need large reducing-power supplementation for the ethanol production. The catabolic pathway of alginate provides both an additional source of sugars and a counterbalance to the excess-reducing equivalents produced by mannitol catabolism, enabling ethanol fermentation from all three sugar components in macroalgae simultaneously (Wargacki et al., 2012). Recently, a protocol for ethanol production using alginate monomers and mannitol as a feedstock has been established with engineered *Saccharomyces cerevisiae*, achieving titers of 4.6% (v/v) (36.2 g/L) (Enquist-Newman et al., 2014). Degradation of alginate polymers into monomers is a critical prerequisite in biofuel processing biotechnologies using alginate (Jagtap et al., 2014; Chaudhary et al., 2018).

Alginate lyase, a polysaccharide lyase (PL), catalyzes alginate degradation through a β-elimination mechanism that forms a double bond between the C4 and C5 of the sugar residue at the non-reducing end (Wong et al., 2000; Jagtap et al., 2014). Thus far, hundreds of alginate lyases have been purified, cloned and characterized from marine bacteria, including *Vibrio*, *Pseudomonas*, and *Azotobacter.* These bacteria commonly contain many alginate lyase genes in their genomes, which always exhibit complementary properties, especially the genera *Vibrio* (Badur et al., 2015; Badur et al., 2017; Chaudhary et al., 2018). These synergistic alginate lyases enable *Vibrio* to efficiently utilize alginate and grow rapidly under a single carbon source (Jagtap et al., 2014; Badur et al., 2015). The Carbohydrate-Active enzymes (CAZy) database lists seven PL families that contain alginate lyases, namely PL 5, 6, 7, 14, 15, 17, and 18^1^. Based on their action mechanism and reaction products, alginate lyases can be further classified into two different action modes: endo-type and exo-type (Wong et al., 2000; Chaudhary et al., 2018; Xu et al., 2018). Thus far, the majority of the reported alginate lyases are classified as endo-type enzymes that degrade alginate polymers into unsaturated alginate oligosaccharides (UAOs) as the final products (Thomas et al., 2013; Li et al., 2016a; Gao et al., 2018). Only a few exo-type alginate lyases (known as oligoalginate lyases) have been reported to degrade alginate polymers and oligomers into monomeric sugar, mainly resulting in unsaturated alginate monosaccharides (UAMs) (Horn et al., 2000; Miyake et al., 2003; Ochiai et al., 2006; Kim et al., 2012; Park et al., 2012; Thomas et al., 2013; Li et al., 2016b; Xu et al., 2017, 2018; Liu et al., 2019). UAMs can transform into 4-deoxy-L-erythro-5-hexoseulose uronic acid (DEH) using not only non-enzymatic process but also the enzymatic process catalyzed by KdgF (Hobbs et al., 2016). DEH was then reduced to 2-keto-3-deoxygluconate (KDG) by a reductase DehR. In some engineered bacteria, KDG can feed into the Entner-Doudoroff (ED) pathway to yield bio-ethanol (Wargacki et al., 2012; Liu et al., 2019). To date, oligoalginate lyases are assigned to four PL families (PL6, 7, 15, and 17) in the CAZy database. PL6 contains three oligoalginate lyases (Li et al., 2016a; Xu et al., 2017; Chaudhary et al., 2018), while PL7, PL15, and PL17 contain one (Thomas et al., 2013), three (Miyake et al., 2003; Ochiai et al., 2006; Jagtap et al., 2014) and nine (Ochiai et al., 2006; Kim et al., 2012; Park et al., 2012; Jagtap et al., 2014; Wang et al., 2015; Yang et al., 2016; Chaudhary et al., 2018; Yu et al., 2018) oligoalginate lyases, respectively. Compared with the extensive research on endo-type alginate lyases, information on the enzymatic properties, biotechnological applications and action mechanisms of oligoalginate lyases is rather rare. Thus far, all the PL17 oligoalginate lyases prefer β-D-mannuronic acid (M) as a substrate; however, the M-preferred substrate recognition and binding mechanism have not been fully elucidated.

In this study, a new PL17 oligoalginate lyase, OalV17, from the marine bacterium *Vibrio* sp. SY01 was cloned and characterized. OalV17 is an exo-type enzyme that yields alginate monomers. Moreover, our results indicates that the residue Arg^231^ in the +1 subsite plays the key role in substrate specificity, which helps to elucidate the M-preferred substrate recognition and binding mechanism of the PL17 family. Furthermore, a rapid and efficient alginate monomer-producing method was developed directly from *L. japonica.*

## Materials and Methods

### Strains and Plasmids

The marine bacterium *Vibrio* sp. SY01 was isolated from Yellow-sea sediment and preserved at the China Center for Type Culture Collection (CCTCC, No. M2018769). *E. coli* strains DH5a and BL21 (DE3), purchased from Solarbio, United States, were grown in Luria-Bertani (LB) medium for plasmid construction and gene expression, respectively. The expression vector pET-22b(+) was purchased from Novagen, United States.

### Sequence Analysis

The genomic sequence of *Vibrio* sp. SY01 was determined and analyzed in our lab. An endo-type alginate lyase, Aly08, has been previously reported (Wang et al., 2019). In this study, a putative oligoalginate lyase-encoding gene, *oalV17*, was identified and cloned from strain SY01 (GenBank accession number: MK689673). The complete *oalV17* open reading frame (ORF) was identified using the ORF finder program. The signal peptide of OalV17 was analyzed by the SignalP 5.1 server. Domain analysis of OalV17 was based on a comparison with the Conserved Domain Database (CDD) of NCBI. To further analyze the theoretical isoelectric point (*pI*) and theoretical molecular weight (Mw) of OalV17, the *pI*/Mw tool was used on the Expasy website^2^. The BLAST algorithm of NCBI was used to search for protein sequences similar to OalV17. Multiple sequence alignment was performed using Clustal X (version 2.1). A phylogenetic tree of OalV17 and other reported oligoalginate lyases was constructed using the bootstrapping neighbor-joining method in MEGA6.

### Expression and Site-Directed Mutagenesis

The gene expression primers for OalV17-EF and OalV17-ER are listed in Supplementary Table S1. Two primers possessed the restriction endonuclease cleavage site and protective base of *Xho*I and *Nco*I at the 5′ ends, respectively. The PCR product and expression vector pET-22b(+) were digested with *Xho*I and *Nco*I restriction endonucleases and purified with gel extraction kits. Next, the digested PCR product was ligated into the vector and then the recombinant plasmid, pET22b(+)-oalV17, was transformed into *E. coli* BL21(DE3) for gene expression. Site-specific mutations in the coding sequence were carried out using the QuikChange site-directed mutagenesis kit (Takara, Dalian, China). The primers used for gene mutation are also listed in Supplementary Table S1.

### Purification of Recombinant OalV17

The *E. coli* BL21(DE3)-pET22b(+)-oalV17 strain was cultured in LB broth and induced at an OD_600_ of 0.6 with 0.1 mM isopropyl-β-thiogalactoside (IPTG) at 20°C and shaking at 200 rpm. After incubation for 16 h, the *E. coli* BL21(DE3)-pET22b(+)-oalV17 strains (100 mL) were harvested by centrifugation (12,000 rpm) at 4°C for 10 min. The recombinant strains were collected and lysed with 20 mL bacterial lysis solution (20 mmol/L Tris-Cl (pH 8.0) with 1 mmol/L EDTA and 20% (w/v) sucrose). Cell debris was removed by centrifugation. The pET22b vector carried an N-terminal pelB signal sequence for potential periplasmic localization of recombinant proteins. However, many activities were also presented in the culture supernatant. Therefore, both of the culture supernatant and lysate were loaded on an Ni-NTA Sepharose column (1 × 5 cm). During the affinity purification protocol, the adsorbed protein was firstly washed with washing buffer (pH 7.6) that contained 500 mM NaCl and 20 mM imidazole in 50 mM phosphate buffer. Next, the target enzyme was eluted with elution buffer (pH 7.6) containing 500 mM NaCl and 100 mM imidazole in 50 mM phosphate buffer. Finally, the purified enzyme was dialysis to remove the NaCl and imidazole. The Mw and purity of the purified OalV17 were analyzed by sodium dodecyl sulfate polyacrylamide gel electrophoresis (SDS-PAGE).

### Biochemical Properties of OalV17

The optimal reaction temperature of OalV17 was determined in 50 mM phosphate buffer (pH 7.2) using temperatures ranging from 0 to 70°C. The optimal pH was determined in 50 mM Britton-Robinson buffer ranging from pH 4 to 11. To determine the thermostability of the enzyme, OalV17 was pre-incubated at different temperatures (0–80°C) for 1 h under 50 mM phosphate buffer (pH 7.2). The activity of the residual enzyme was then determined at its optimal temperature and pH. To analyze the pH stability for OalV17 activity, the enzyme solution was pre-incubated in 50 mM Britton-Robinson buffer (pH 4–11) at 4°C for 12 h. Then, the residual activity was measured under normal assay conditions (50 mM phosphate buffer, pH 7.2). The effects of different metal ions and detergents on the enzymatic activity of OalV17 were measured in the presence of various compounds (1 mM) that were added to the substrate solution. To determine the metal ion and detergent resistance of OalV17, the enzyme solution was incubated with different concentrations of metal ions (10–40 mM; Fe^3+^, Zn^2+^, Ca^2+^, and Mg^2+^) or detergents (1–10 mM; Tween 20, Tween 80, SDS and TritonX-100) for 24 h. The residual activity of the enzyme solution was then determined under normal assay conditions. Un-incubated enzyme was used as a control.

### Enzymatic Activity Assay

Sodium alginate (20–50 kDa, 100–260 monosaccharides in a polymer, M/G ratio: 1.66) was purchased from Bright Moon Seaweed Group, Qingdao, China. To determine the enzymatic activity of OalV17, 900 μL of 0.3% (w/v) sodium alginate solution (pH 7.2) in 50 mM phosphate buffer was used as the substrate and pre-incubated at 40°C for 5 min. Then, 100 μL of enzyme solution was added into the substrate and incubated at 40°C for 10 min. The reaction was quantified using the 3, 5-dinitrosalicylic acid (DNS) method at OD_520_ (Chaudhary et al., 2018). Glucuronic acid (Sigma-Aldrich, United States) and used as to produce the standard curve. One unit of activity was defined as the amount of enzyme that release 1 μmol of reducing sugar per minute under the above conditions.

### Substrate Specificity and Kinetic Parameters

Alginate contains two different uronic acids, namely α-L-guluronic acid (G) and β-D-mannuronic acid (M) (Senturk Parreidt et al., 2018). For use in this study, polyG and polyM blocks (20-25 monosaccharides in a polymer, purity: 95%), were purchased from Qingdao BZ Oligo Biotech Co., Ltd. Qingdao, China. To determine the substrate specificity of OalV17, three different substrates [specifically 0.3% (w/v) sodium alginate, polyG blocks and polyM blocks in 20 mM phosphate buffer, pH 7.2] were digested with OalV17 solution at 40°C for 10 min. These three substrates were also used to determine the kinetic constants of OalV17 and the different single mutants. The kinetic constants were measured at varying substrate concentrations from 0.1 to 8 mg/mL (Li et al., 2016a). Lineweaver-Burk plots were created and analyzed using a standard linear regression function to obtain the apparent kinetic parameters (*K*_m_ and *V*_max_). Using polyM blocks and polyG blocks as substrates, unsaturated alginate disaccharides (DM and DG, respectively) were prepared from the degradation products of AlySY08 from *Vibrio* sp. SY08 (Li et al., 2016a). The reaction production of DM and DG (10 mg/mL) were degraded by OalV17 (0.1 mg) for 1 h and analyzed by thin-layer chromatography (TLC) on an HPTLC plate (Merck, Germany) and developed with *n*-butanol/formic acid/water (2:1:1, by vol). The TLC plate was visualized using a diphenylamine/aniline/phosphate reagent after drying and coloring at 80°C for 30 min (Hu et al., 2013).

### Molecular Modeling and Docking Analysis

The 3D-structure of the receptor protein (oligoalginate lyase OalV17) was built by homology modeling using the Modeller 9.18 package. The crystal structure of oligoglainete lyase Alg17c from *Saccharophagus degradans* 2-40 (PDB ID: 4NEI_A) was chosen as the template (Park et al., 2014). The protein structure possessing the best DOPE score was chosen as the final homology model. The ligand (DM or DG) was drawn by ChemDraw. AutodockTool was then used to determine the atom types and calculate the atom charges of the receptor protein, and the pdbqt file was saved for docking (Trott and Olson, 2010). Finally, Pymol was used to analyze the docking solutions and to construct graphical presentations and illustrative figures.

### Reaction Product Analysis

Action modes and reaction products of OalV17 were analyzed using sodium alginate (3 mg/mL) as the substrate. The OalV17 solution (1 mL, 20 U) was mixed with substrate (10 mL) and incubated at 40°C for 30 min. The reaction products were analyzed by TLC. The reaction products were further analyzed on the ÄKTA avant 150 platform (GE Health, United States) using a Superdex peptide 10/300^TM^ column (GE Health) at 235 nm (Li et al., 2016b; Chaudhary et al., 2018). Ammonium bicarbonate (0.2 M) was used as the mobile phase. The column pressure was limited to 1.5 MPa and the flow rate was 0.6 mL/min. After ethanol precipitation, the final products were determined by negative ion electrospray ionization mass spectrometry (ESI-MS) as previously described (Chaudhary et al., 2018).

### Enzymatic Saccharification of *L. japonica*

Enzymatic saccharification of *L. japonica* was mainly performed according to Li et al. (2019). Briefly, natural sun-dried *L. japonica* with a feed ratio of 1:7 (w/v) was pretreated with cellulase (Celluclast 1.5 L, Novozyme A/S, Denmark), with a dry weight of 3%, for releasing fermentable sugars. Next, the engineered yeast strain *Yarrowia lipolytica* with alginate lyase activity was grown in an algae-based medium. After fermentation for 72 h, glucose and mannitol were completely consumed, mainly resting alginate oligosaccharides (DP 2-3) (Li et al., 2019). Next, the supernatant of *E. coli* BL21(DE3)-pET22(+)-oalV17 strains (containing approximately 100 mg/L OalV17) was added into the reaction system at a ratio of 1:10 (∼10 mg/L OalV17 in the final system). After reaction for 1 h, the yeast strain consumed the residual nutrients and was then removed by centrifugation. The reaction system continued to operate at room temperature for 12 h. The reaction was analyzed using TLC chromatography. The final reaction products were also analyzed by SE-HPLC with a superdex peptide 10/300^TM^ column. The total sugar content of the reaction products was measured by the phenol-sulfuric acid method, using D-glucose as a standard. The glucose and mannitol concentrations were determined using the Glucose (HK) Assay Kit and D-Mannitol Colorimetric Assay Kit (Sigma-Aldrich, United States), respectively.

## Results

### Sequence Analysis of OalV17

**FIGURE 1:**
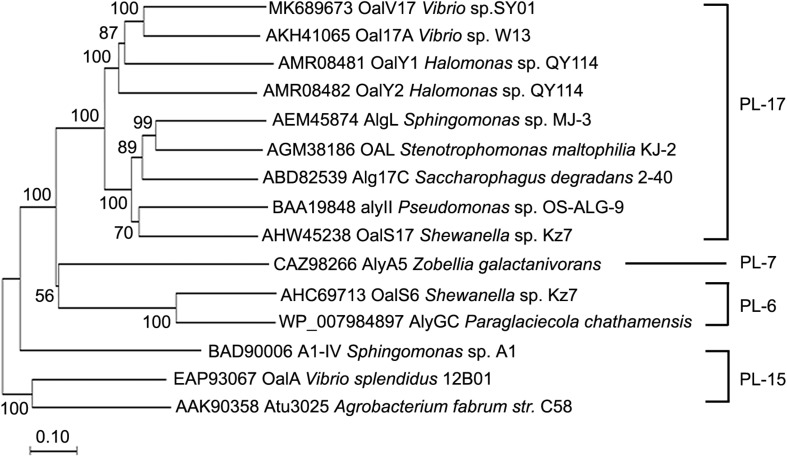
Phylogenetic analysis of OalV17 and other reported oligoalginate lyases. ClustalX 2.1 was used to align the protein sequence. Phylogenetic tree was constructed by the neighbor-joining method using MEGA 6.0. The reliability of the phylogenetic reconstructions was determined by boot-strapping values (1500 replicates). Branch-related numbers are bootstrap values (confidence limits) representing the substitution frequency of each amino acid residue.

In our previous study, the marine bacterium *Vibrio* sp. SY01 was isolated from Yellow Sea sediment and grown rapidly in alginate sole-carbon medium (Wang et al., 2019). In this study, the oligoalginate lyase-encoding gene, *oalV17*, was cloned from this strain. This gene contains 2,163 bp in the ORF and the encoding protein, OalV17, is composed of 720 amino acid residues. Signal peptide analysis showed that OalV17 is not predicted to have a signal peptide at its N-terminus. Additionally, the theoretical *pI* and Mw of OalV17 are 5.3 and 81.5 kDa, respectively. According to the CDD search on NCBI, OalV17 is a new oligoalginate lyase with a single-domain belonging to the PL17. A phylogenetic tree was constructed containing OalV17 and other reported oligoalginate lyases (Figure 1). A deep branching clade was found in the phylogenetic tree between OalV17 and Oal17A from *Vibrio* sp. W13 (Genbank number AKH41065), which belongs to PL17. Moreover, the protein identity between OalV17 and Oal17A is only 46.8%, which indicates that OalV17 is a new enzyme.

**FIGURE 2:**
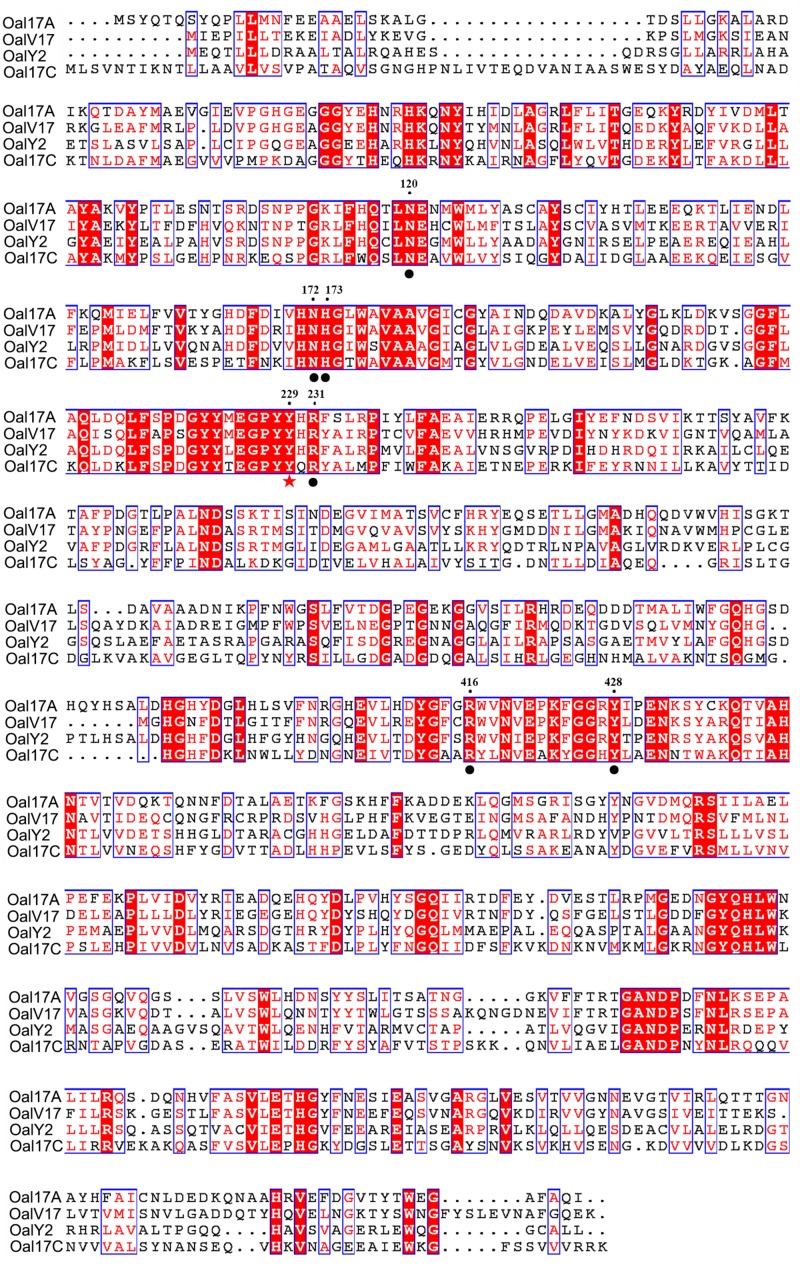
Sequence comparison of OalV17 with related oligoalginate lyases from PL family 17. The conserved substrate binding sites and catalytic sites are marked with circle and star, respectively.

In order to verify conserved amino acids, OalV17, Oal17A, OalY2, and Alg17c [the typical PL17 oligoalginate lyase (Park et al., 2014)] were analyzed by multiple sequence alignment. The catalytic amino acid (Tyr^229^) and carbohydrate binding amino acids (Asn^120^, Asn^172^, His^173^, Arg^231^, Arg^416^, and Tyr^428^) of PL17 were completely conserved in the OalV17 sequence (Figure 2). The protein model was successfully constructed with 100% confidence. The overall architecture of OalV17 consists of two domains: an amino-terminal imperfect β-barrel and a carboxyl-terminal α-sheet domain (Supplementary Figure S1A), which is similar to the crystal structure of Alg17c. Co-crystals of Alg17c show that Tyr^258^ and Tyr^450^ are the active sites. These results indicate that OalV17 is a new member in PL17.

### Purification and Biochemical Characterization of OalV17

**FIGURE 3:**
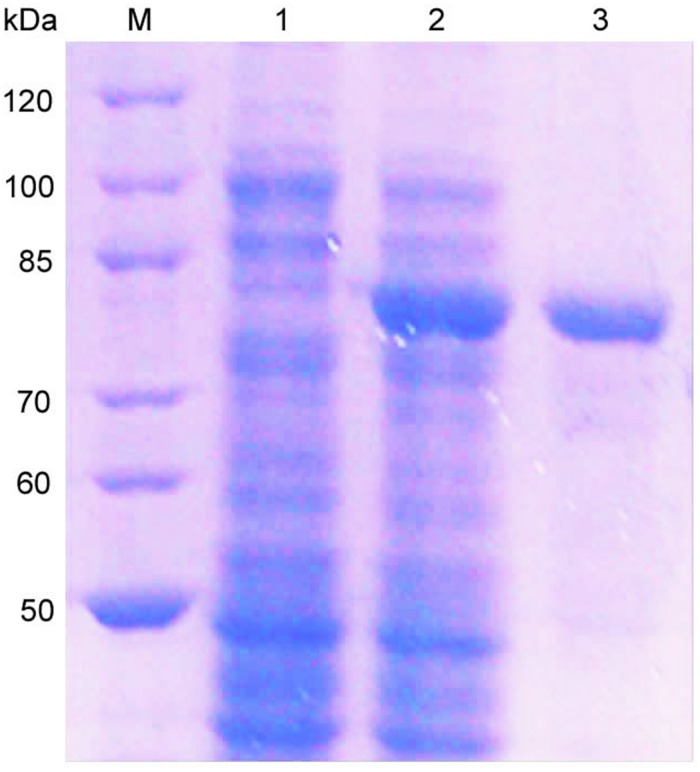
SDS-PAGE analysis of the recombinant enzyme. *Lane* M: marker; *Lane* 1: recombinant bacteria without IPTG; *Lane* 2: recombinant bacteria induced with IPTG; *Lane* 3: the purified OalV17 with His-tag affinity chromatography.

The *oalV17* gene was over-expressed in *E. coli* BL21(DE3) through the pET22b(+) plasmid. The recombinant OalV17 enzyme was purified using one-step affinity Ni-NTA Sepharose column chromatography, with a yield of 71.6% and a concentration of approximately 68.7 mg/L. SDS-PAGE analysis indicates that the Mw of OalV17 is approximately 82 kDa (Figure 3), which in agreement with its theoretical Mw (81.5 kDa). The specific activity of OalV17 was 33.9 U/mg, as determine by the DNS method, which is a common method to detect oligoalginate lyase activity using colorimetric absorption (Chaudhary et al., 2018; Jiang et al., 2019; Vuoristo et al., 2019). The specific activity of OalV17 was 2.18 U/mg, as determined by TBA method, which is another common detection method for alginate lyase activity.

**FIGURE 4:**
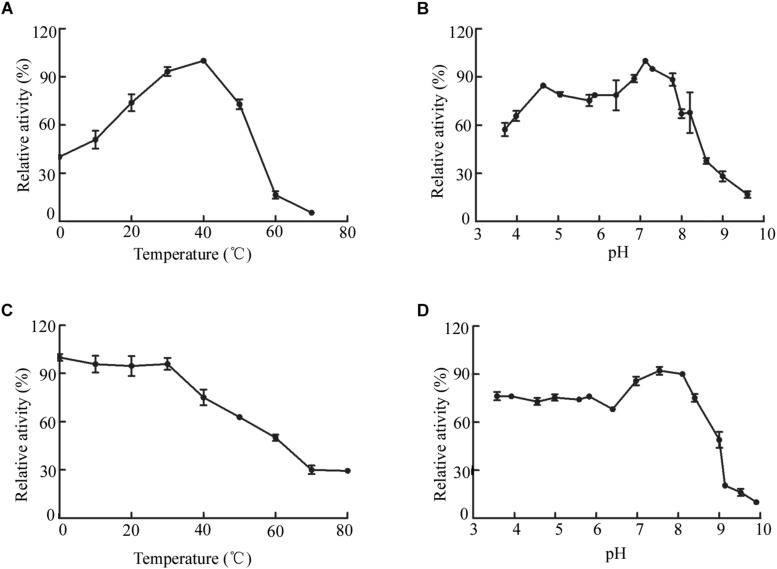
Biochemical characteristics of OalV17. **(A)** The optimal temperature of OalV17. **(B)** The optimal pH of OalV17. **(C)** The thermal-stability of OalV17. **(D)** The pH stability of OalV17. The data were expressed as mean ± SD, *n* = 3. The activity of control (100% relative activity) is 10.8 U/ml.

The biochemical properties of OalV17 were determined using the purified enzyme. The optimal reaction temperature and pH of OalV17 were 40°C and 7.2, respectively (Figures 4A,B). As shown in Figure 4C, 74.5, 61.2, and 47.6% of the enzymatic activity remained after incubation for 1 h at 40, 50, and 60°C, respectively. Additionally, OalV17 showed a relatively wide pH range, retaining over 70% of its initial activity after incubation for 12 h at pH 3.4–8.0 (Figure 4D). Other characterized oligoalginate lyase AlgL17, from *Microbulbifer* sp. ALW1, was stable at 25°C, but not stable at 30 and 35°C while it showed stability at a narrow pH range of 5.0–8.0 (Jiang et al., 2019). Additionally, AlgC-PL7, isolated from *Cobetia* sp. NAP1, showed thermostability and salt-tolerance but this kind of oligoalginate lyase strongly inhibited by metal ions (Cu^2+^) (Yagi et al., 2016). The enzymatic activity in the presence of metal ions and chemical reagents was measured using various mental compounds at a concentration of 1 mmol/L. Although the enzymatic activity of OalV17 was not dependent NaCl, its addition significantly increased the activity (Supplementary Table S2). All the tested divalent metal ions, including Cu^2+^, Zn^2+^, Mg^2+^, Ni^2+^, Ba^2+^, Mn^2+^, and Ca^2+^, increased OalV17 activity; however, the trivalent metal ions Al^3+^ and Fe^3+^ inhibited OalV17 activity. The chelating agent, EDTA, strongly inhibited enzymatic activity, indicating that OalV17 activity depends on divalent metal ions (Supplementary Table S2). Another oligoalginate lyase Oal17A, from *Vibrio* sp. W13, exhibited inhibition effects toward most of the divalent ions and chelating agent (Yu et al., 2018).

The resistance of OalV17 to metal ions and detergents was determined after incubation of the enzyme for 24 h in different concentration of metal ions or detergents. The activity of OalV17 was greater than 90% after incubation with divalent metal ions, including Zn^2+^, Mg^2+^, and Ca^2+^ (Table 1). Moreover, OalV17 retained more than 70% activity at different concentrations of trivalent metal ions (Fe^3+^). These results indicate that OalV17 is a metal ion-resistant enzyme, which will enable it to perform stably during the processing of brown seaweed. The detergent-resistance of OalV17 was also determined after incubation for 24 h in various surfactants, including Tween-20, SDS, Tween-80 and Triton X-100 (from 1–10 mM). Thus, OalV17 showed surfactant-resistant properties, retaining more than 77% activity after incubation with different surfactants (Table 1). To the best of our knowledge, similar properties have not yet been reported for the known oligoalginate lyases.

**TABLE 1:** Metal ion- and detergent-resistance of OalV17.

Metal ion incubation	Concentration (mM)	Relative activity (%)	Detergent incubation	Concentration (mM)	Relative activity (%)
CaCl_2_	10	99.1 ± 6.5	SDS	1	95.8 ± 6.1
	20	92.1 ± 7.1		5	85.7 ± 3.1
	40	91.5 ± 7.3		10	77.5 ± 4.9
ZnCl_2_	10	93.8 ± 2.5	Tween 20	1	86.3 ± 8.5
	20	92.9 ± 9.6		5	85.2 ± 8.1
	40	90.0 ± 3.1		10	78.5 ± 6.1
MgCl_2_	10	93.4 ± 4.2	Tween 80	1	92.8 ± 1.5
	20	91.9 ± 9.6		5	88.3 ± 4.3
	40	90.4 ± 1.1		10	85.4 ± 10.3
FeCl_3_	10	79.7 ± 4.5	TritonX-100	1	85.4 ± 6.0
	20	77.9 ± 1.8		5	84.7 ± 9.4
	40	76.3 ± 6.1		10	81.4 ± 14.1

*The data were expressed as mean ±SD, n = 3. The enzyme un-incubated in any reagents was used as a control. The activity of control (100% relative activity) is 10.8 U/ml.*

### Substrate Specification and Kinetic Parameters of OalV17 and Its Mutants

Thus far, all of the reported PL17 oligoalginate lyases exhibit a preference for polyM blocks. Herein, three different alginate polymeric substrates, namely sodium alginate, polyM blocks and polyG blocks, were used to study the substrate specificity of OalV17. Our results indicate that OalV17 also prefers polyM blocks (81.6 ± 6.1 U/mg) over polyG blocks (15.1 ± 2.7 U/mg) and sodium alginate (33.9 ± 2.3 U/mg).

**FIGURE 5:**
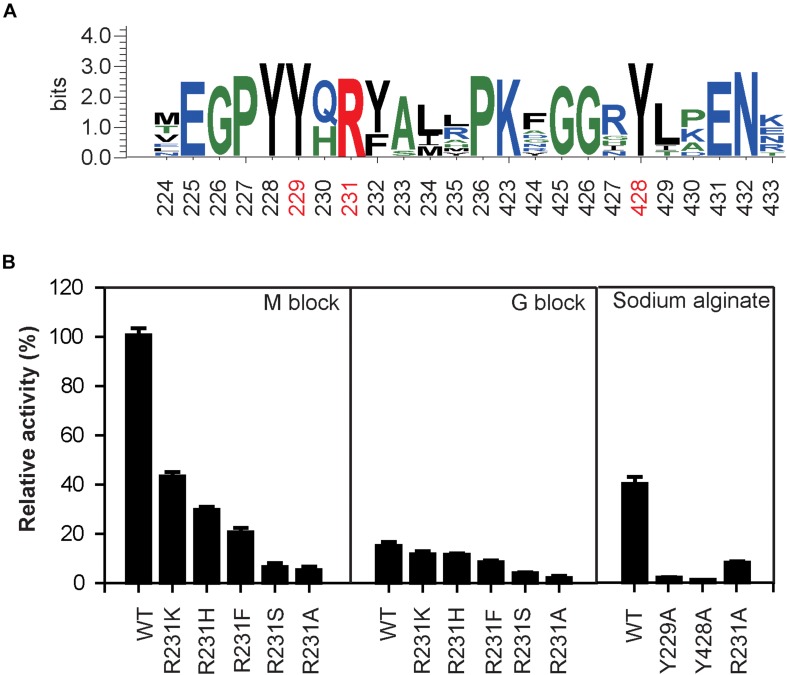
Sequence logo analysis of PL17 family **(A)** and relative activity of different mutations in different substrate **(B)**. The data were expressed as mean ± SD, *n* = 3. The activity of control (100% relative activity) is 17.2 U/ml.

The sequence logo of the active site residues in the PL17 family of oligoalginate lyases indicate that the residues located inside the catalytic cavity are highly conserved (Figure 5A). Consistent with docking analysis and the β-elimination mechanism of PL17 oligoalginate lyases, the role of the conserved Tyr^229^ is a general base and Tyr^428^ is a general acid in OalV17. To determine the importance of the catalytic residues Tyr^229^ and Tyr^428^, we mutated them into alanine. The subsequent specific activity assays indicated that the single Y229A and Y428A mutants were both completely devoid of activity, which further indicates that residues Tyr^229^ and Tyr^428^ are the active sites of OalV17 (Figure 5B).

Docking analysis indicates that residue Arg^231^ in subsite +1 may play an important role in the binding of M/G (Supplementary Figure S1C). The residue Arg^231^ was also completely conserved in PL17 (Figure 5A). Thus, five site-directed mutations (R231K, R231H, R231F, R231S, and R231A) were used to determine the importance of this residue for substrate specificity. When Arg^231^ was mutated to Ala, there was a 92.1% diminution of specific activity using sodium alginate as a substrate. For further investigation of its role, Arg^231^ was replaced with the phenyl- and hydroxyl-containing residues His, Lys, Ser and Phe. The mutations of R231K, R231H and R231F resulted in significant loss of activity toward polyM blocks, but little change toward polyG blocks (Figure 5B).

Subsequently, the apparent kinetic parameters of OalV17 and its mutants toward polyM blocks and polyG blocks were calculated using the Lineweaver–Burk formula. The determination of Michaelis constant (*K*_m_) indicates that OalV17 has a higher affinity value toward polyM blocks (0.40 mg/mL) than polyG blocks (1.98 mg/mL). The site-directed mutations of Arg^231^ induced drastic changes in substrate affinity (Table 2). Compared to the wild type, the R231K, R231H, and R231F mutations caused a significant reduction in substrate affinity for polyM blocks. However, those mutations did not induce a drastic change in affinity for polyG blocks. These results further indicate that Arg^231^ plays an important role in substrate specification.

**TABLE 2:** Kinetic parameters of OalV17 and its mutants.

Substrates	Mutants	*K*_m_ (mg/ml)	*V*_max_ (U/mg)
PolyM blocks	Wide type	0.40 ± 0.09	146.4 ± 7.6
	R231K	1.05 ± 0.31	100.5 ± 2.7
	R231H	0.80 ± 0.24	71.3 ± 14.1
	R231F	1.78 ± 0.04	64.8 ± 2.5
	R231S	2.75 ± 0.94	43.4 ± 10.5
	R231A	2.89 ± 0.91	38.3 ± 7.9
PolyG blocks	Wide type	1.98 ± 0.71	60.2 ± 8.5
	R231K	2.01 ± 0.16	57.6 ± 3.6
	R231H	1.99 ± 0.13	53.8 ± 2.9
	R231F	2.46 ± 0.34	37.1 ± 3.9
	R231S	2.79 ± 0.73	20.7 ± 7.1
	R231A	2.91 ± 0.23	27.3 ± 3.7

### Reaction Products of OalV17

**FIGURE 6:**
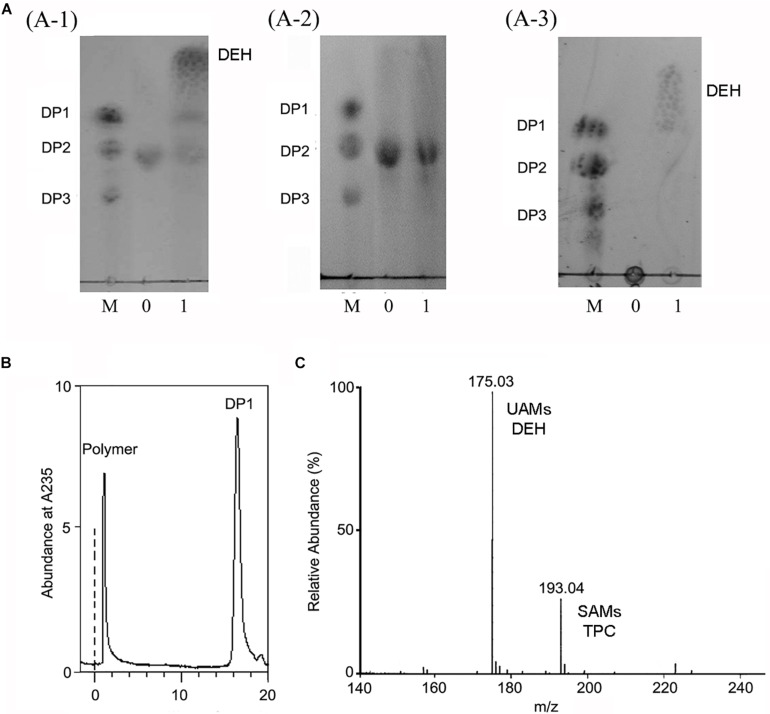
Reaction product of OalV17. **(A)** TLC analysis of degradation product of different substrate (**A-1**, DM substrate; **A-2**, DG substrate; **A-3**, sodium alginate substrate). Line M, standard alginate oligosaccharides; Line 0, substrate; Line 1, reaction product. **(B)** SE-HPLC analysis of reaction product using sodium alginate as substrate. **(C)** Negative ions ESI-MS of product using sodium alginate as substrate. DP1 represents unsaturated alginate monosaccharide, DEH represents non-enzymatically transformation of unsaturated alginate monosaccharide, DP2 represents unsaturated alginate disaccharide, DP3 represents unsaturated alginate trisaccharide.

The alginate oligosaccharide reaction products of OalV17 were analyzed using DM and DG as substrates, respectively. TLC analysis showed that OalV17 could efficiently degrade DM (Figure 6A-1). However, the catalytic activity toward DG was much weaker (Figure 6A-2). These results indicate that OalV17 can recognize alginate disaccharides as the minimal substrate, with preference for DM as a substrate.

Sodium alginate polymer was used to determine the action type of OalV17. As shown in Figure 6A-3, only the monomers were detected on the TLC plates, while the intermediates were not found under the same conditions. The reaction products were also determined by SE-HPLC using the Superdex peptide 10/300^TM^ column. Only alginate polymer and monosaccharides (DP1) were observed at the absorbance of 235 nm (Figure 6B). As documented previously, oligoalginate lyases degrade both alginate polymers and oligosaccharides, mainly into UAMs and saturated alginate monosaccharides (SAMs). The UAMs can non-enzymatically convert to DEH under dynamic equilibrium. In our preliminary study, we found that DEH could further transform into 2,4,5,6-tetrahydroxytetrahydro-2-pyran-2-carboxylic acid (TPC) automatically, which were two cyclic hemiacetal stereoisomers formed by predominantly hydration of the DEH molecules (Li et al., 2016a). Enquist-Newman et al. also showed that DEH molecules in the oligoalginate lyases-degrading products were predominantly hydrated to form two cyclic hemiacetal stereoisomers. In this study, the final products were determined with negative ion ESI-MS following ethanol precipitation to remove the undegraded alginate polymer. As shown in Figure 6C, the main peaks were 175.03 *m/z* and 193.04 *m/z.* The 175.03 *m/z* corresponded to UAMs and DEH (Park et al., 2012). The molecular weight of SAMs and TPC were 194 which is corresponded the peak of 193.04 *m/z* at negative ion ESI-MS analysis. This result further indicates that OalV17 yields alginate monomers as the main reaction products.

### Optimization of Alginate Monomeric Sugar Acid Production From *L. japonica*

**FIGURE 7:**
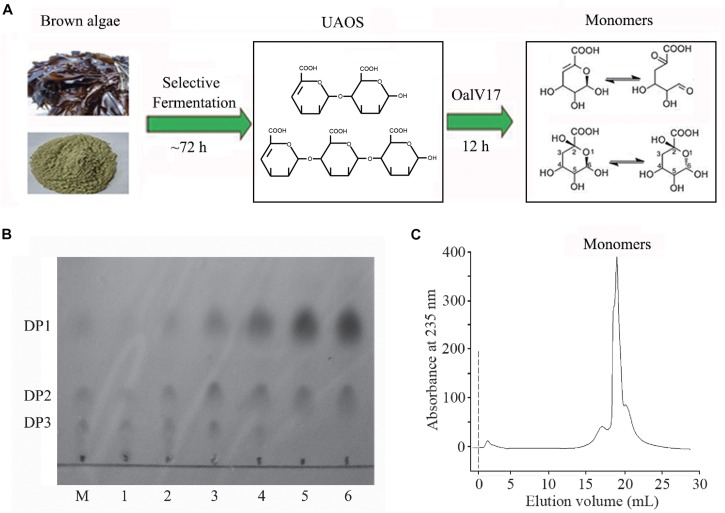
Scheme of degrading protocol **(A)**, TLC **(B)**, and SE-HPLC **(C)** analysis of the products from *L. japonica*. **(A)** The scheme of degrading protocol. **(B)** TLC analysis of the product. M, standard UAOs marker; 1, Yeast growth for 72 h; 2, Adding OalV17 for 1 h; 3, Adding OalV17 for 3 h; 4, Adding OalV17 for 6 h. 5, Adding OalV17 for 12 h; 6, Adding OalV17 for 24 h. **(C)** SE-HPLC analysis of degradation product of OalV17 for 12 h.

In our previous study, a rapid and efficient alginate oligosaccharide-producing protocol using *L. japonica* was developed by combining enzymatic hydrolysis and fermentation (Li et al., 2019). In this study, we optimized and developed a protocol to produce alginate monomeric sugar acid directly from *L. japonica* by combining oligoalginate lyase OalV17 with our previous technique (Figure 7A). Along with yeast growth and degradation, alginate polymers were firstly degraded into alginate oligosaccharides (Figure 7B) and then glucose and mannitol were completely consumed through the biological elimination of the engineer yeast. With the addition of oligoalginate lyase OalV17, the oligosaccharides were further degraded to alginate monomeric sugar acids. The final products were further determined by SE-HPLC (Figure 7C). Almost all oligosaccharides were degraded to monosaccharides. Thus, our research indicates that OalV17 is an good candidate for alginate saccharification and biofuel production.

## Discussion

The marine bacterium *Vibrio* sp. SY01 grew rapidly in alginate sole-carbon medium (Wang et al., 2019). In this study, an oligoalginate lyase-encoding gene, *oalV17*, was cloned from this strain. Commonly, alginate degrading bacteria contain 2 or 3 oligoalginate lyase genes within their genomes (Jagtap et al., 2014; Chaudhary et al., 2018). In the CAZy database, two putative PL17 oligoalginate lyase genes co-located in the genome are commonly found, especially the genus *Vibrio*. These genes usually have a synergistic degradation effect. For example, OalB and OalC from *V. splendidus* have complementary properties (Jagtap et al., 2014). Among the reported oligoalginate lyases, PL6 enzymes prefer polyG substrates, while PL17 enzymes prefer polyM substrates (Chaudhary et al., 2018). Interestingly, the putative PL17 and PL6 genes are commonly co-located in bacterial genomes. In our previous study, a combination of OalC6 (PL6) and OalV17 (PL17) displayed a synergistic degradation ability for both alginate polymers and oligomers (Chaudhary et al., 2018). However, the genome of *Vibrio* sp. SY01 contained only one putative oligoalginate lyase gene, *oalV17*. When we cloned the upstream and downstream regions of this gene, no putative oligoalginate lyase genes were found through sequence analysis.

In this study, an adequate biochemical characterization of OalV17 was conducted. Our results indicate that OalV17 has a higher specific activity (33.9 ± 2.3 U/mg) toward alginate than other characterized oligoalginate lyases, such as OalA, OalB and OalC from *V. splendidus* 12B01 (28.5 U/mg, 20 U/mg and 20.8 U/mg, respectively); A1-IV (16.1 U/mg) from *Sphingomonas* sp. A1; Aly17C (1.98 U/mg) from *Saccharophagus degradans* 2-40; and OalS6 and OalS17 (33.7 U/mg and 32 U/mg, respectively) from *Shewanella* sp. Kz7 (Ochiai et al., 2006; Kim et al., 2012; Park et al., 2012; Jagtap et al., 2014; Wang et al., 2015; Yang et al., 2016; Chaudhary et al., 2018; Yu et al., 2018). In this study, we found that several metal ions could enhance the activity of OalV17 and we also showed that OalV17 can resist high concentrations of many metal ions and detergents (Table 1). To the best of our knowledge, similar properties have not yet been reported among the known oligoalginate lyases. These properties make OalV17 a good candidate for biotechnological and industrial applications for brown algae processing.

Thus far, all of the oligoalginate lyases in PL17 prefer polyM blocks as a substrate. However, the M-preferred substrate recognition and binding mechanism of this family have not been fully elucidated. In this study, site-directed mutagenesis followed by kinetic analysis showed that the residue Arg^231^ plays a key role in substrate specificity. When Arg^231^ was mutated to Ala, the R231A mutation lost almost all of its activity, indicating that R231 in oligoalginate lyase OalV17 is an essential residue. The docking analysis indicates that residue Arg^231^ in subsite +1 may play an important role in the binding of M/G (Supplementary Figure S1C). To further investigate its important role in substrate specification, R231 was replaced with phenyl- and hydroxyl-containing residues Phe and Ser, respectively. These mutants resulted in significant loss of activity toward polyM blocks, thereby indicating that both the hydroxyl group and the phenyl ring are key elements in substrate recognition. R231 was further replaced by His and Lys, which contain positively charged amino groups, spatially substituting the hydroxyl of tyrosine. Furthermore, the *K*_m_ values for those mutants apparently increased toward the polyM blocks (Table 2), demonstrating that the mutations reduced the enzyme activities by weakening the binding of substrates to the enzyme. Although these mutations also reduced the reaction activities and binding abilities toward polyG blocks, the trend was not obvious, especially for the R231K and R231H mutations. Altogether, these results indicate that the guanidyl group of Arg231 residue plays a critical role in substrate specification of OalV17.

As previously documented, there are many reports of oligoalginate lyases producing alginate momomers using sodium alginate as a substrate (Miyake et al., 2003; Ochiai et al., 2006; Thomas et al., 2013; Li et al., 2016a; Xu et al., 2017; Vuoristo et al., 2019). Alginate lyases with different modes of action and substrate specification could have a synergistic degradation capability (Wang et al., 2014; Chaudhary et al., 2018). However, an efficient protocol to obtain alginate monomeric sugar acids directly from brown seaweed is lacking. Alginate accounts for 22–44% of the dry weight of brown seaweed. However, traditional bioethanol producing strains, such as *Saccharomyces cerevisiae*, *Zymobacter palmae*, and *Pichia angophorae*, cannot directly use alginate polymers as an energy source. Thus, bioethanol production from raw brown seaweed is low, as the alginate remains unutilized (Horn et al., 2000; Yarden and Sliwkowski, 2001; Liu et al., 2019). In our previous study, a rapid and efficient alginate oligosaccharide-producing protocol directly from *L. japonica* was developed by combining enzymatic hydrolysis and a fermentation procedure (Li et al., 2019). Many divalent metal ions (mainly Ca^2+^) are released during the processing of brown algae (Ahmady-Asbchin et al., 2009). Furthermore, during pre-treatment processing, certain detergents are used to wash brown algae. The residual metal ions and detergents disturb the activity of related degrading enzymes (Hifney et al., 2018). Therefore, the stability of the enzymes in the presence of metal ions and surfactant agents are important characteristics for their industrial application. In this study, we further developed and optimized a protocol to produce alginate monomeric sugar acid directly from *L. japonica* by combining the oligoalginate lyase OalV17 with our previous technique.

## Conclusion

In this study, a new oligoalginate lyase, OalV17, from the marine bacterium *Vibrio* sp. SY01 was cloned and characterized. OalV17 is an exo-type enzyme that yields alginate monomers as products. Moreover, our results indicate that the residue Arg^231^ in the +1 subsite plays a key role in substrate specificity, which helped elucidate the M-preferred substrate recognition and binding mechanism of the PL17 family. This study demonstrates that the combined properties of OalV17, such as a high catalytic efficiency and stability, as well as resistance to metal ions and detergents, are superior to the properties of previously characterized enzymes, making it a good candidate for further theoretical study and biotechnological use.

## Data Availability Statement

The raw data supporting the conclusions of this article will be made available by the authors, without undue reservation, to any qualified researcher.

## Ethics Statement

This article does not contain any studies with human participants or animals performed by any of the authors.

## Author Contributions

SL, NH, and M-SL designed the experiments. SL, NH, and LW performed the research and analyzed the data. NH performed the statistical analysis. SL, BL, and SJ performed the sequence analysis *in silico*. SL and NH directed the research analyzed the data. All authors wrote the manuscript.

## Conflict of Interest

The authors declare that the research was conducted in the absence of any commercial or financial relationships that could be construed as a potential conflict of interest.

## Footnotes

1 http://www.cazy.org/

2 https://www.expasy.org/

## Supplementary Material

FIGURE S1 The overall structure and molecular docking analysis of OalV17 and substrates (DM and DG). **(A)** The overall three-dimensional structure of OalV17. **(B)** Molecular docking analysis of OalV17 and alginate disaccharides. **(C)** Analysis of binding amino acid residues with DM and DG, respectively.

TABLE S1 Primers used in this study.

TABLE S2 Effect of metal ions and EDTA on the activity of OalV17.
