# Supplementary figures and images for: Biochemical Characterization of a New Oligoalginate Lyase and Its Biotechnological Application in Laminaria japonica Degradation
Source: Front Microbiol. 2020 Mar 10;11:316. doi: 10.3389/fmicb.2020.00316 (PMC7076127; doi:10.3389/fmicb.2020.00316)

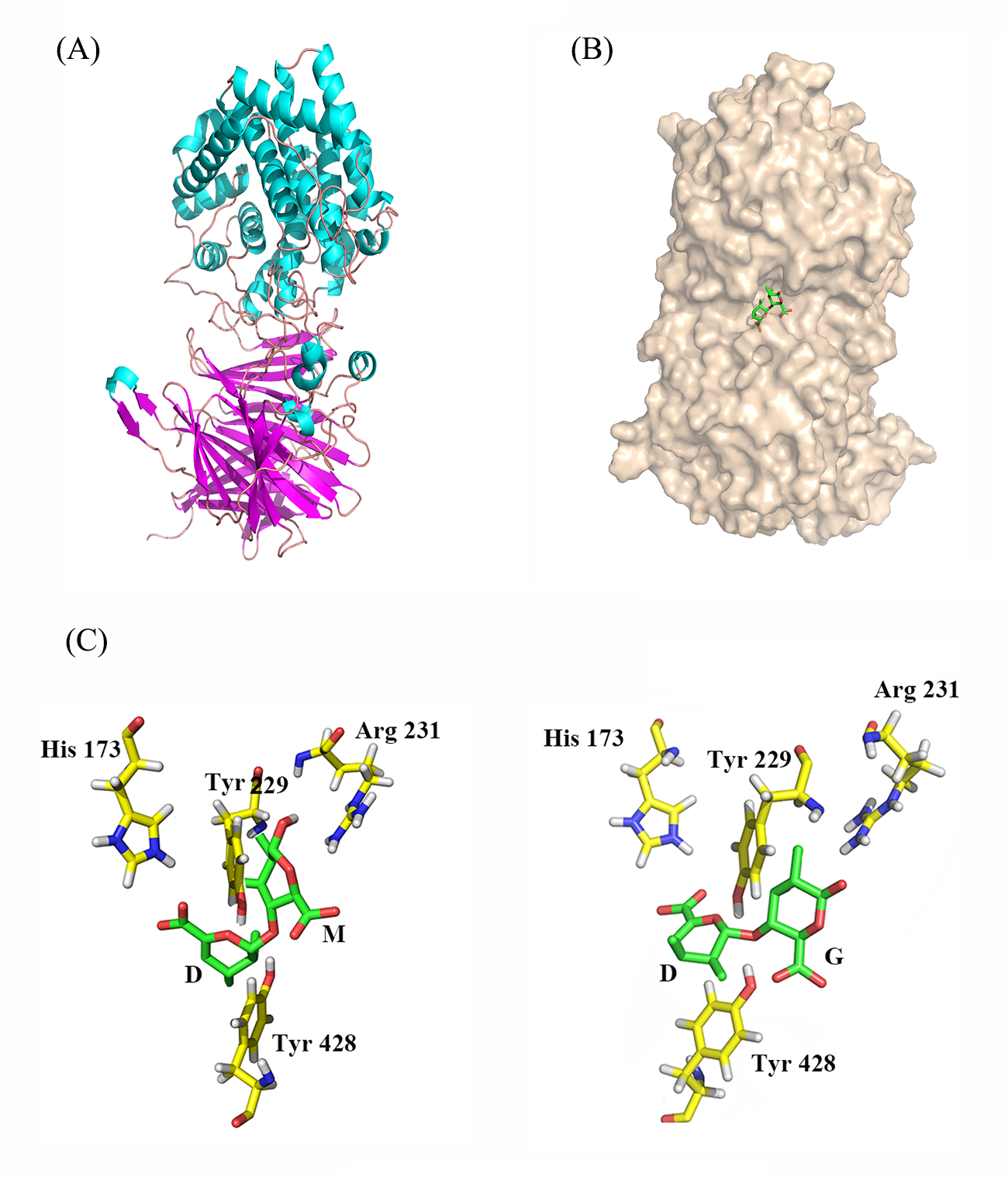

Supplement: FIGURE S1 — The overall structure and molecular docking analysis of OalV17 and substrates (DM and DG). (A) The overall three-dimensional structure of OalV17. (B) Molecular docking analysis of OalV17 and alginate disaccharides. (C) Analysis of binding amino acid residues with DM and DG, respectively. [file Image_1.tif]
