# Supplementary material for: Biochemical Characterization of a New Oligoalginate Lyase and Its Biotechnological Application in Laminaria japonica Degradation
Source: Front Microbiol. 2020 Mar 10;11:316. doi: 10.3389/fmicb.2020.00316 (PMC7076127; doi:10.3389/fmicb.2020.00316)
Supplement: TABLE S1 — Primers used in this study. [file Table_1.doc]

**Table S1** Primers used in this study

| Primers | Sequence (5' to 3') |
| --- | --- |
| OalV17-EF | *CATG* CCATGG ATG ATC GAA CCT ATT CTA TT |
| OalV17-ER | *CCG* CTCGAG TTT CTC CTG CCC GAA TGC GT |
| Y229A-F | ATG GAA GGT CCG TAC GCA CAT CGT TAT GCA ATT C |
| Y229A-R | TAC CTT CCA GGC ATG CGT GTA GCA ATA CGT TAA G |
| Y428A-F | AAA TTC GGC GGT CGT GCC CTA GAC GAA AAC AAA |
| Y428A-R | TTT AAG CCG CCA GCA CGG GAT CTG CTT TTG TTT AG |
| R231A-F | GGT CCG TAC TAC CAT GCA TAT GCA ATT CGT CCA A |
| R231A-R | CCA GGC ATG ATG GTA CGT ATA CGT TAA GCA GGT T |
| R231K-F | GGT CCG TAC TAC CAT AAA TAT GCA ATT CGT CCA A |
| R231K-R | CCA GGC ATG ATG GTA TTT ATA CGT TAA GCA GGT T |
| R231H-F | GGT CCG TAC TAC CAT CAT TAT GCA ATT CGT CCA A |
| R231H-R | CCA GGC ATG ATG GTA GTA ATA CGT TAA GCA GGT T |
| R231F-F | GGT CCG TAC TAC CAT TTT TAT GCA ATT CGT CCA A |
| R231F-R | CCA GGC ATG ATG GTA AAA ATA CGT TAA GCA GGT T |
| R231S-F | GGT CCG TAC TAC CAT AGC TAT GCA ATT CGT CCA A |
| R231S-R | CCA GGC ATG ATG GTA TCG ATA CGT TAA GCA GGT T |
