# Supplementary material for: Biochemical Characterization of a New Oligoalginate Lyase and Its Biotechnological Application in Laminaria japonica Degradation
Source: Front Microbiol. 2020 Mar 10;11:316. doi: 10.3389/fmicb.2020.00316 (PMC7076127; doi:10.3389/fmicb.2020.00316)
Supplement: TABLE S2 — Effect of metal ions and EDTA on the activity of OalV17. [file Table_2.doc]

**Table S2 Effect of metal ions and EDTA on the activity of OalV17**

| Reagent  added | Concentration  (mM) | Relative  activity (%) | Reagent  added | Concentration  (mM) | Relative  activity (%) |
| --- | --- | --- | --- | --- | --- |
| None | -- | 100±1.8 | CaCl_2_ | 1 | 112.1±3.5 |
| NaCl | 1  10 | 103.9±3.9  109.3±2.1 | CuCl_2_  ZnCl_2_ | 1  1 | 108.1±3.3  114.1±3.8 |
|  | 100 | 120.8±0.4 | BaCl_2_ | 1 | 109.4±4.2 |
|  | 200 | 129.7±0.1 | MnCl_2_ | 1 | 120.7±3.1 |
|  | 300 | 126.9±1.9 | MgCl_2_ | 1 | 106.2±0.7 |
|  | 500 | 125.8±2.2 | CoCl_2_ | 1 | 110.4±2.4 |
| KCl | 1 | 103.0±4.6 | FeCl_3_ | 1 | 86.6±5.1 |
| NH_4_Cl | 1 | 101.4±2.4 | AlCl_3_ | 1 | 49.9±8.7 |
| LiCl | 1 | 94.4±2.6 | EDTA | 1 | 15.8±0.6 |

The data were expressed as mean ± SD, n=3. The activity of control (100% relative activity) is 10.8 U/ml.
